# LncRNA NRON promotes the proliferation, metastasis and EMT process in bladder cancer

**DOI:** 10.7150/jca.37958

**Published:** 2020-01-17

**Authors:** Tiefu Xiong, Chenchen Huang, Jianfa Li, Shaokang Yu, Fangfang Chen, Zeng Zhang, Chengle Zhuang, Yawen Li, Changshui Zhuang, Xinbo Huang, Jing Ye, Fangting Zhang, Yaoting Gui

**Affiliations:** 1 Graduate School, Guangzhou Medical University, Guangzhou 510182, China; 2 Guangdong and Shenzhen Key Laboratory of Male Reproductive Medicine and Genetics, Institute of Urology, Peking University Shenzhen Hospital, Shenzhen 518036, China; 3 Department of Oncology, Peking University Shenzhen Hospital, Shenzhen 518036, China; 4 Anhui Medical University, Hefei 230000, Anhui Province, China

**Keywords:** long non-coding RNA, lncRNA NRON, bladder cancer, oncogene, EMT

## Abstract

*Background*: Bladder cancer (BC) is one of the most common malignancies world-wide with high morbidity and mortality. Long noncoding RNAs (lncRNAs) are thought to play a critical role in cancer development. LncRNA NRON, a repressor of activated T-cell nuclear factor (NFAT), has been shown to be dysregulated in many cancer types. However, the clinical significance and molecular mechanism of NRON in bladder cancer is still unknown.

*Methods*: The expression levels of NRON in BC tissues and cell lines were tested by RT-qPCR. Survival analysis was performed to detect the correlation between NRON expression and clinical outcomes in patients with BC. The biological role of NRON in BC cells proliferation and metastasis was examined *in vitro* and *in vivo*.

*Results*: The expression of NRON was significantly upregulated in BC specimens and cell lines compared with paired adjacent normal tissues and normal cell lines. The upregulation of NRON in bladder cancer patients was significantly associated with the depth of bladder tumor invasion and poor prognosis. Knockdown of NRON inhibited BC cells proliferation, migration, invasion and tumorigenicity. Furthermore, NRON promoted epithelial-mesenchymal transition (EMT) progression, and NRON-induced EZH2 expression contributed to this process.

*Conclusion*: In conclusion, our results suggested that NRON acted as an oncogene and tumor biomarker for BC.

## Introduction

Bladder cancer (BC) is one of the most common malignancies world-wide with high morbidity and mortality, bringing huge economic burden. Over 50% of patients relapse within 6-12 years after initial diagnosis 1-3. It is well known that surgery, radiotherapy and chemotherapy are major treatments for BC patients. However, the therapeutic effects of these recommended treatments are still limited, and the five-year overall survival rate has remained at low level in the past decades 4-7. Recently, numerous biomarkers associated with the development and prognosis of BC has been discovered, but few have eventually been applied to clinical practice. Therefore, early diagnostic markers and novel therapeutic targets are urgently required to cure BC.

Long non-coding RNAs (lncRNA) are defined as transcripts longer than 200 nucleotides (nts) without obvious protein coding potential 8, 9. LncRNAs can bind with various proteins, DNA or RNA to form functional complexes that are involved in multiple cellular processes, including epigenetic modification 10, 11, transcription 12, 13, post-transcriptional regulation 14-16, signal transduction 17, and many more. In post-transcriptional regulation, LncRNAs can act as competing endogenous RNAs (ceRNAs) by interacting with miRNAs to influence the stability of their targets 10, 18, 19. Their function is usually determined by their sequence, structure and location 20. LncRNAs are involved in physiological and pathological processes such as cancer progression^10^, organ development 14, immune regulation 21, stem cell maintenance^22^ and pathogen infection 23, 24. In addition, LncRNAs are specifically expressed in certain types of cancer and can be detected in blood or urine 25, 26. LncRNAs is a new type of potential biomarkers and therapeutic targets for cancer treatment.

More and more lncRNA have been shown to act as oncogenes or tumor suppressors in the initiation and development of cancers 27, 28. For instance, HOTTIP has been reported to act as oncogenes in various cancers, including hepatocellular carcinoma, gastric cancer, colorectal cancer, pancreatic cancer, lung cancer, prostate cancer and osteosarcoma 29. Zhou, et al. 30 and Ying, et al. 31 illustrated that MEG3 was downregulated in multiple tumor types and inhibited cell proliferation, acting as a tumor suppressor. Several lncRNA have been reported to serve as oncogenes in BC, such as H19, MALAT1, TUG1, UCA1 32-35, emphasizing the potential for lncRNA to serve as biomarkers and therapeutic targets in BC.

The activated T-cell nuclear factor (NFAT), a family of transcription factors, was first identified in the nucleus extracts of activated T cells 36. The *NFAT* family consists of 5 members (*NFAT1*, *NFAT2*, *NFAT3*, *NFAT4* and *NFAT5*), playing critical roles in cancer invasion, migration and angiogenesis 37. LncRNA NRON (NRON) acts as a suppressor of NFAT by inhibiting nucleocytoplasmic shuttling of NFAT. NRON inhibited the proliferation and invasion of vascular endothelial cells through reducing *NFAT* nucleus transfer 38. In human disease other than cancer, it has been reported that NRON modulates HIV-1 transcription and replication by increasing NFAT activity 39. Subsequently, NRON was shown to contribute to HIV-1 latency by inducing specifically degradation of tat protein 40. NRON attenuates atrial fibrosis by promoting NFATc3 phosphorylation 41. NRON has also been shown to contribute CMV-enhanced CD28^null^ CD8^+^ T cell aging by modulating NFAT signaling 42. Interestingly, NRON also regulates the circadian clock via regulating PER and CRY subcellular translocation 43.

In human cancers, it has been reported that NRON was down-regulated in hepatocellular carcinoma (HCC) and overexpression of NRON can suppress HCC growth and metastasis 44, 45. NRON was also down-regulated in triple-negative breast cancer (TNBC), and NRON down-regulates lncRNA snaR to inhibit TNBC cell proliferation 46. Although many studies about lncRNA NRON have been reported, the role and underlying mechanisms of NRON in BC is still unknown.

In this study, we showed that the expression of NRON was increased in BC tissues, and NRON up-regulation was significantly associated with the depth of bladder tumor invasion and poor prognosis in patients with BC. We also found that knockdown of NRON inhibited malignant phenotypes of BC cells, including proliferation, migration, invasion and tumorigenicity. Furthermore, NRON upregulation promoted epithelial-mesenchymal transition (EMT) progression, and NRON-induced EZH2 expression contributed to this process. Our results suggested that NRON acted as an oncogene and tumor biomarker for BC.

## Materials and methods

### Sample collection

In this study, we collected 42 pairs of BC tissues and adjacent normal bladder tissues from the patients who underwent BC tissues resection at Peking University Shenzhen Hospital (Shenzhen, Guangdong, China). This project was approved by the Ethics Committee of Peking University Shenzhen Hospital, China. The clinical and pathological characteristics of patients were recorded and summarized. All specimens were immediately dipped in RNAlater® RNA Stabilization Reagent (Qiagen GmbH, Hilden, Germany) after the operation and then stored in -80 ℃ refrigerators.

### Cell lines and cell cultures

All cell lines were obtained from the American Type Culture Collection (Manassas, VA). Cell lines were maintained using standard media and conditions. Specifically, human BC cells (J82, 5637, T24, UMUC3, SW780) and human normal bladder epithelial cell (SV-HUC1) were maintained in Roswell Park Memorial Institute (RPMI) 1640, Dulbecco's modified Eagle's medium or F-12K (Gibco; Thermo Fisher Scientific. Inc, Waltham, MA, USA) supplemented with 10% fetal bovine serum, 1% penicillin-streptomycin and maintained at 37°C supplied with 5% CO2 atmosphere.

### Cell transfection

Cells were transfected with siRNAs or negative control (si-NC) using Lipofectamine 3000 (Invitrogen, Carlsbad, CA) at 70~80% confluence in 6-well plates. Cells were harvested 48 hours after transfection. The sequence of si-NRON was: 5'-GAGUUGGAGGUGUUGAAGCAAAUAU-3'. The si-NRON and si-NC were purchased from GenePharma (Suzhou, China).

### RNA extraction, cDNA synthesis and RT-qPCR

Total RNAs were extracted with the TRIzol reagent (Invitrogen; Thermo Fisher Scientific, Inc. Waltham, MA, USA) according to the manufacturer's instructions. The cDNA was synthesized with random primers using a reverse transcription kit PrimeScript RT reagent Kit (Takara Biomedical Technology, Dalian, China). RT-qPCR was performed on the Roche Lightcycler 480 Real-Time PCR system (Roche Diagnostics, Basel, Switzerland) with the SYBR Premix Ex Taq kit (Takara Biomedical Technology). GAPDH was chosen as the internal control. The expression level of NRON in tissues and cells was analyzed using the 2-ΔΔCq method. The primer sequences were as follows: NRON primers forward: 5′- AGCCCAAGCTTCACATCTCTAATGTAAACAACCCAGC -3′ and reverse: 5′- CGGGGTACCGGAAAAAATTTCTCCTTAACTATTTC -3′. GAPDH primers forward: 5′- GGTATGACAACGAATTTGGC -3′, reverse: 5′-GAGCACAGGGTACTTTATTG-3′.

### Cell counting kit 8 (CCK-8) assay

After transfection, 3×10^3^ cells were plated in 96-well culture plates. The absorbance in each well was measured at 0, 24, 48 and 72 hours by a microplate reader (Bio Rad Laboratories, Inc. Hercules, CA, USA), 60 min after adding the CCK-8 kit (Dojindo, Kumamoto, Japan) in the dark at 37°C and a humidified incubator containing 5% CO2.

### 5-ethynyl-20-deoxyuridine assay (EdU) Assay

EdU assay was carried out by using EdU assay kit (Ribobio, Guangzhou, China) in 5637 and SW780 cells following manufacturer's protocol. Images were detected and recorded with a microscope at 200 × (Olympus, Tokyo, Japan). Original cells released blue fluorescence and proliferating cells released green fluorescence under the fluorescent microscopy. The evaluation index of cell proliferation activity was the ratio of EdU-stained cells (with green fluorescence) to Hoechst-stained cells (with blue fluorescence).

### Wound healing assay

The ability of cell migration was examined using wound healing assay. 5637 and SW780 cells were transfected with si-NRON or si-NC in 6-well culture plates for 48 hours, which allowed cells to grow to 80-90% confluence. A bio-clean 0.2 ml pipette tip was used to draw vertical lines. After washed with phosphate buffer saline (PBS), the cells were incubated with serum-free medium for 24 hours. The area of the scratch was photographed and recorded by a digital camera system (Olympus Corporation, Tokyo, Japan) at 0 hour and 24 hours in 4 fields.

### Transwell assay

Transwell assay without or with Matrigel was used to examine BC cell migratory and invasive ability according to manufacturer's instructions. BC cells (5637 and SW780) were transfected with siRNA in 6-well culture plate for 48 hours. After then, BC cells were collected and resuspended with serum-free medium. Control and invasion chambers (BD Biosciences, Franklin Lakes, NJ, USA) were placed into 24-well culture plates containing complete RPMI1640 or DMEM culture medium. Then, 5×10^4^ cells resuspended in culture media with Optin-MEM were added into control or invasion chambers without or with Matrigel. After incubating for 36 hours (migration) or 48 hours (invasion), cells at the bottom of the chamber were fixed with 4% paraformaldehyde (30 min) and stained with 0.5% crystal violet (30 min). Migrated and invasive cells were photographed and quantified in 3 randomly selected fields of view using a light microscope.

### Flow cytometry assay

Both BC cell lines (5637 and SW780 cell) were transfected with si-NRON or si-NC in 6-well culture plate for 36 hours. At 36 hours, cells were treated with Annexin V-fluorescein isothiocyanate and propidium iodide (Invitrogen; Thermo Fisher Scientific, Inc.) according to manufacturer introductions. After 15min of staining at room temperature in darkness, 0.3ml of binding buffer was added to each prepared tube. The apoptosis rate was measured with a FACSCalibur flow cytometer (BD Biosciences).

### *In vivo* animal model

All animal procedures were performed with the approval of the Animal Care and Use Committee of Peking University Shenzhen Hospital, China. For tumor growth assays, 2 × 10^6^ cells were suspended in 0.1 ml PBS and subcutaneously implanted into the flank of male BALB/c-Nude mice (aged 4-5 weeks). Tumor growth was examined twice a week and tumor volume was estimated by the formula LW^2^ / 2, where L is the length and W is the width of the tumor. Mice were euthanized 4 weeks after tumor cell implantation and tumor growth was measured.

### Western blot analysis

BC cells (5637 and SW780) and tumor tissues from animal experiments were collected, and cell lysates were prepared using 10% sodium dodecyl sulfate lysis buffer and protein concentration was quantified using a BCA protein assay kit (Thermo Fisher Scientific, CA, USA). Proteins (20 ul per lane) were separated by 10% SDS-PAGE and transferred to PVDF membranes (Millipore, Billerica, MA, USA). Immunoblotting of the membranes using the following primary antibodies: anti-EZH2, anti-E-cadherin, anti-N-cadherin, anti-Vimentin and anti-GAPDH (Abcam, China). After incubation with primary antibodies and secondary antibody, the chemiluminescent signal was detected. The scanned images were quantified using ImageJ software. The experiments were repeated at least three times.

### Statistical analysis

Data were presented as mean ± standard deviation and the Student's t-test analysis of variances were used to statistically analyze using SPSS 23.0 (IBM SPSS, Chicago, IL, USA) statistical software in this study. All experiments we performed *in vitro* were repeated at least three times with samples in triplicates. Group difference was assessed using Student's t-test. Survival analysis curves were calculated using the Kaplan-Meier method and statistically compared by log-rank test. In this study, *P* < 0.05 was considered as statistically significant (* *P* < 0.05, ** *P* < 0.01, *** *P* < 0.001).

## Results

### NRON is upregulated in BC tissues

**Figure 1:**
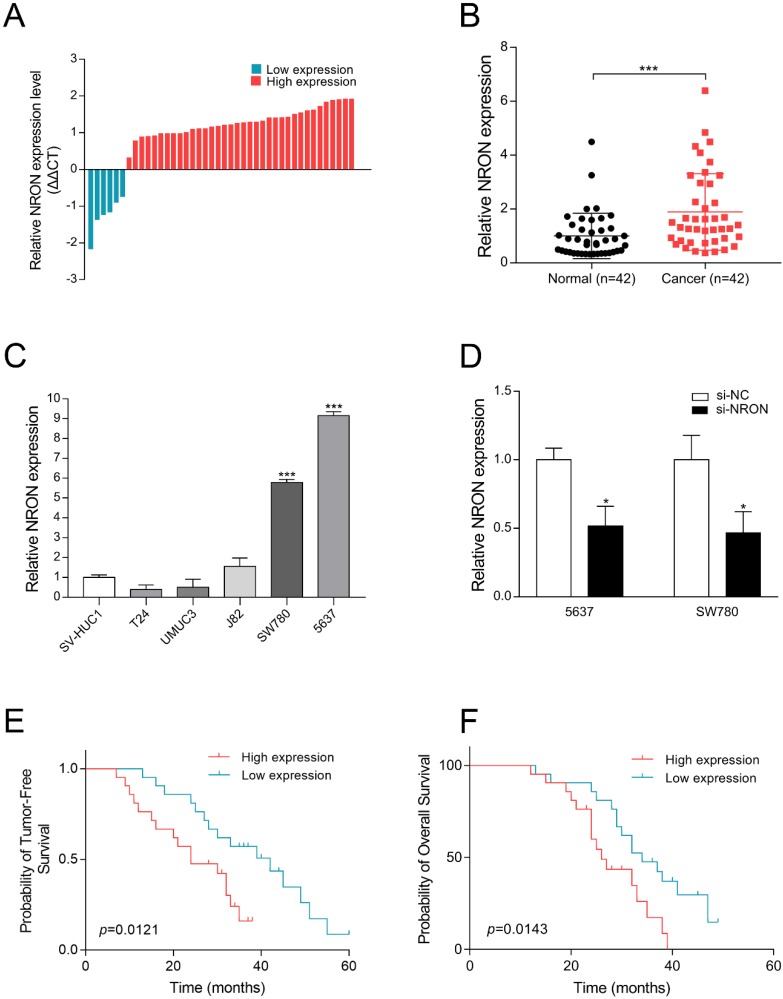
NRON is upregulated in bladder cancer (BC) tissues and cell lines. (A, B) RT-qPCR analysis of the relative expression levels of NRON in 42 pairs of BC tissues (Cancer) and matched adjacent normal tissues (Normal). (C) The relative expression levels of NRON in BC cell lines compared to SV-HUC1 cell line. (D) Relative expression levels of NRON in BC cells (5637 and SW780) transfected with si-NRON (*P* < 0.05). (E, F) Survival curves of OS and RFS. Patients were grouped into NRON-Low or NRON-High based on the NRON expression level. The means ± SD from 3 independent repeated experiments. *P < 0.05, **P < 0.01, ***P < 0.001.

To investigate the potential involvement of NRON in the tumorigenesis of human BC, the expression levels of NRON in BC tissues and matched nonneoplastic tissues from 42 patients were analyzed by RT-qPCR. The results showed that the expression of NRON was up-regulated in 85.7% (36/42) of human BC tissues compared with paired adjacent normal tissues (Fig. 1A and 1B). The mean expression of NRON in BC tissues was significantly higher than that in adjacent normal tissues (Fig. 1B, n = 42, *P* < 0.001).

In cell lines, NRON expression was significantly increased by 9.16-fold in 5637 cell and 5.79-fold in SW780 cell (Fig. 1C, *P* < 0.05) compared to SV-HUC1 (normal uroepithelium cell line). No significant difference was observed in other BC cell lines (T24, UMUC3, J82). Therefore, 5637 and SW780 cells were chosen for further experiments.

### Upregulation of NRON is significantly associated with poor survival of patients with BC

We further analyzed the correlations between NRON expression in BC tissues and the tumor invasion depth of BC patients. Table 1 displayed that NRON upregulation was significantly associated with BC tumor invasion depth (*P* < 0.05), but gender, age, tumor size and TNM stage were independent of NRON expression levels (Table 1). These results indicate that NRON may play an oncogenic role in BC.

Moreover, Kaplan-Meier survival analysis revealed that patients with high NRON expression had a significantly worse recurrence-free survival (RFS, P = 0.0121, Fig. 1E) and overall survival (OS, P = 0.0143, Fig. 1F) than those with low expression. Taken together, our results revealed that NRON is upregulated in BC, and NRON expression levels could serve as an independent predictor of prognosis in patients with BC.

### NRON promotes BC cells proliferation

These above findings promoted us to explore the role of NRON in tumorigenesis. We first designed small interfering RNAs (siRNA) that specifically targeted NRON, and test the efficacy of siRNA by RT-qPCR. Compared with the control (si-NC treatment), the mRNA level of NRON in si-NRON transfected cells was reduced by 48.3% in 5637 cells, and decreased by 53.2% in SW780 cells (Fig. 1D, *P* < 0.05).

**Figure 2:**
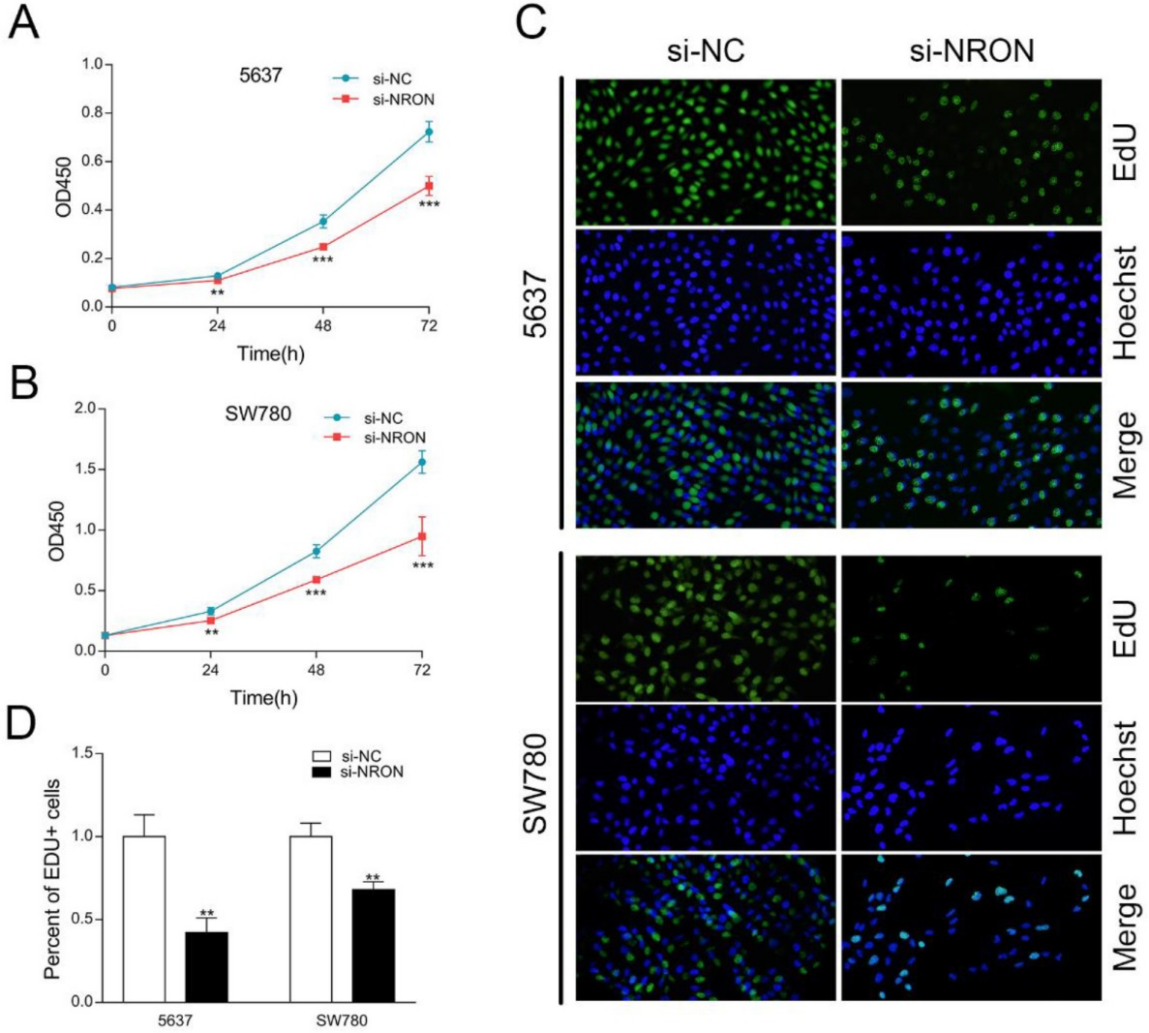
NRON promotes cell proliferation. (A and B) The proliferation rate of 5637 and SW780 cells transfected with si-NRON or si-NC measured by CCK-8 assay. (C and D) EdU assay was used to assess the proliferative abilities of 5637 and SW780 cells transfected with si-NRON or si-NC. The means ± SD from 3 independent repeated experiments. *P < 0.05, **P < 0.01, ***P < 0.001.

CCK-8 and EdU assays were carried out to test whether NRON knockdown inhibited the proliferation of 5637 and SW780 cells. As shown in results of CCK-8 assay, NRON knockdown in 5637 and SW780 cells markedly attenuated cell proliferation (Fig. 2A and B). Similar results were observed in EdU assays (Fig. 2C and D). These results suggest that NRON knockdown inhibits BC cells proliferation.

### NRON promotes BC cells migration, invasion and EMT process

**Figure 3:**
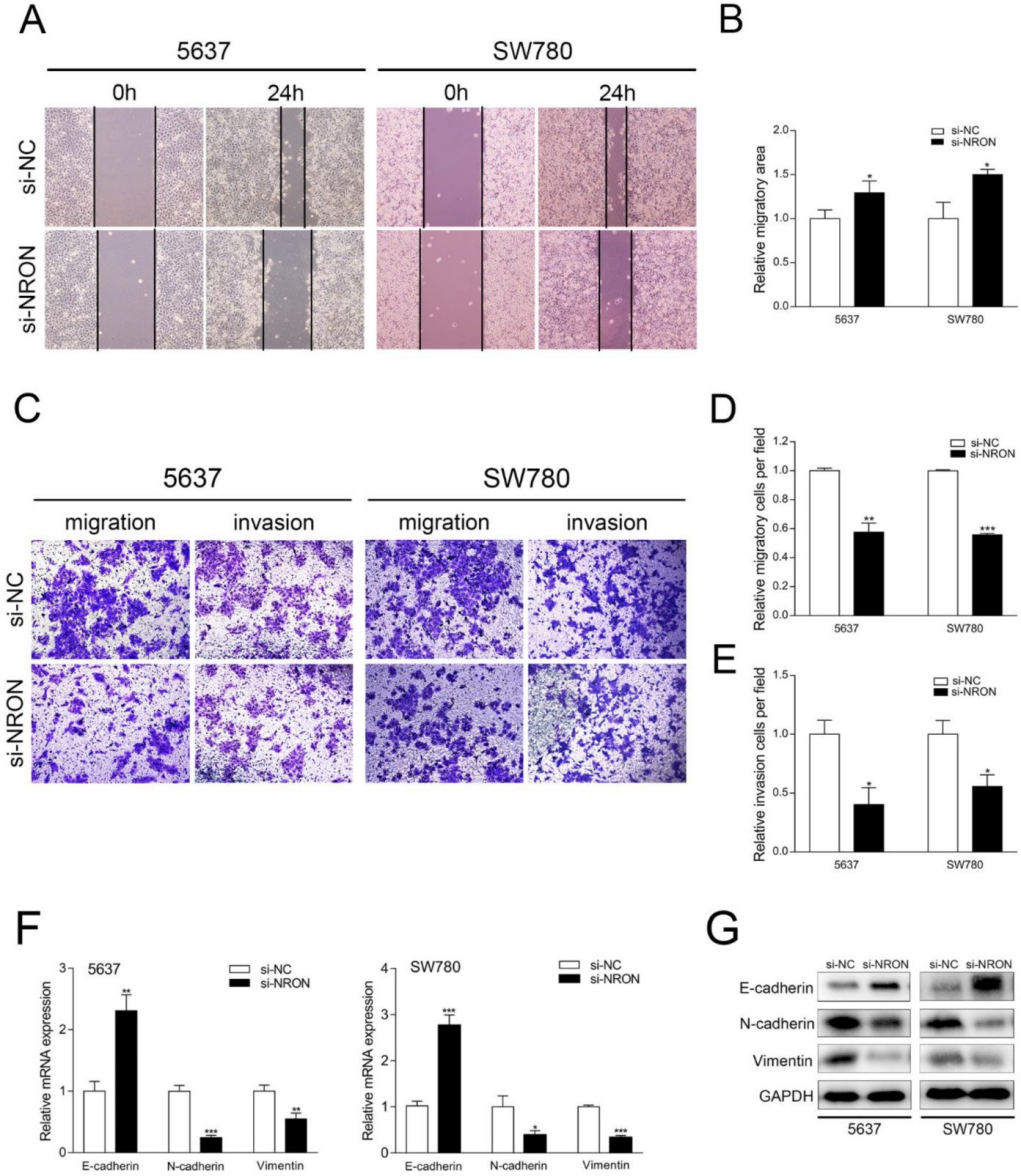
NRON promotes cell migration. (A) Representative images of wound healing assays of 5637 and SW780 cells transfected with si-NRON or si-NC. Magnification, 200×. (B) Quantification of relative migration of 5637 and SW780 cells transfected with si-NRON or si-NC. (C) Representative images of transwell assay of 5637 and SW780 cells transfected with si-NRON or si-NC. Magnification, 200×. (D, E) Quantification of relative migration and invasion of 5637 and SW780 cells transfected with si-NRON or si-NC. (F, G) The expression of EMT markers were detected using RT-qPCR and western blotting in BC cell lines (5637 and SW780). The means ± SD from 3 independent repeated experiments. *P < 0.05, **P < 0.01, ***P < 0.001.

Wound healing assay and transwell assay (without Matrigel coating) were utilized to assess the impact of NRON in BC cell migration. Wound healing assay revealed that the open wound area of si-NRON transfected cells was significantly larger than that of si-NC transfected cells 24 hours after scratching treatment (Fig. 3A and B). The fold change was 1.29-fold in 5637 cells and 1.50-fold in SW780 cells (Fig. 3A and B). Transwell assay showed that the numbers of migrated cells in si-NRON treated groups were significantly decreased than that in si-NC treated groups. The fold change was 42.4% in 5637 cells and 44.3% in SW780 cells (Fig. 3C and 3D).

The effects of NRON on BC cell invasion were tested by transwell assay with Matrigel. We found that si-NRON transfection significantly inhibits the invasion of 5637 and SW780 cells (Fig. 3E). The number of invaded cells in si-NRON group was reduced by 59.8% in 5637 cells and by 44.3% in SW780 cells (Fig. 3E, P < 0.05). These results indicate that NRON promotes cell migration and invasion.

Emerging evidence implicates that EMT enables the cancer cells to acquire mesenchymal phenotype and metastasize towards distant sites. To determine whether NRON regulates EMT of BC cells, we detected the expression levels of EMT markers (E-cadherin, N-cadherin and Vimentin) by RT-qPCR and Western blot. Knockdown of NRON resulted in increased expression of E-cadherin, and decreased expression of N-cadherin and vimentin in BC cells (Fig. 3F-3G). These data indicate that NRON promotes cell migration, invasion and EMT progression of BC cells.

### The apoptotic rate of BC cell lines is no statistically affected by NRON knockdown

**Figure 4:**
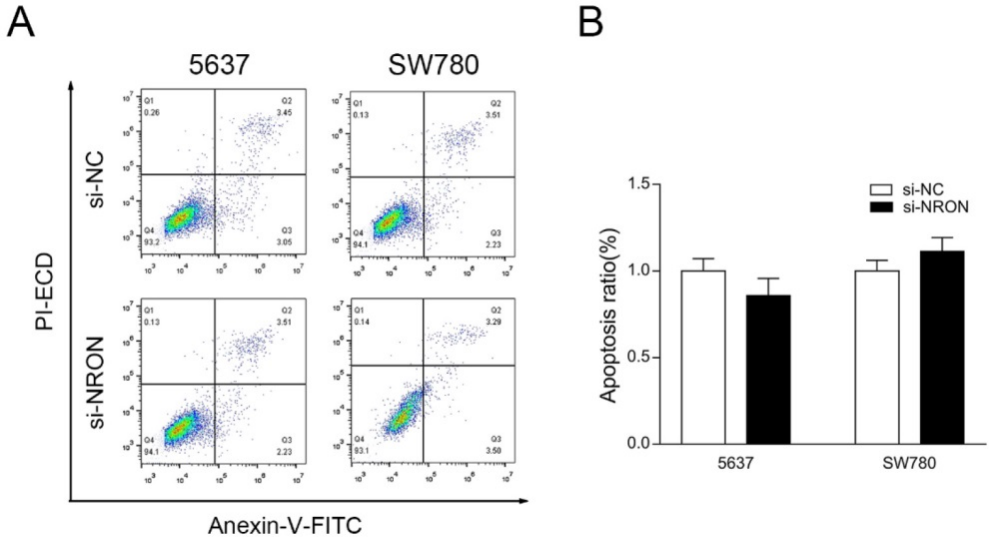
Knockdown of NRON has no statistically influence on the apoptotic rate of BC cells. (A and B) Representative dot plots of Annexin V/PI staining of BC cells transfected with indicate siRNA. Quantification of BC cells apoptosis. The means ± SD from 3 independent repeated experiments. *P < 0.05, **P < 0.01, ***P < 0.001.

Flow cytometry assay was performed to test whether NRON knockdown can promote BC cells apoptosis. However, the apoptotic rate between the 5637 cells transfected with si-NRON or si-NC was not statistically significant (*P* > 0.05, Fig. 4A and B). Similar results were observed in SW780 cells (*P* > 0.05, Fig. 4A and B).

### NRON promotes tumorigenicity of BC cells

**Figure 5:**
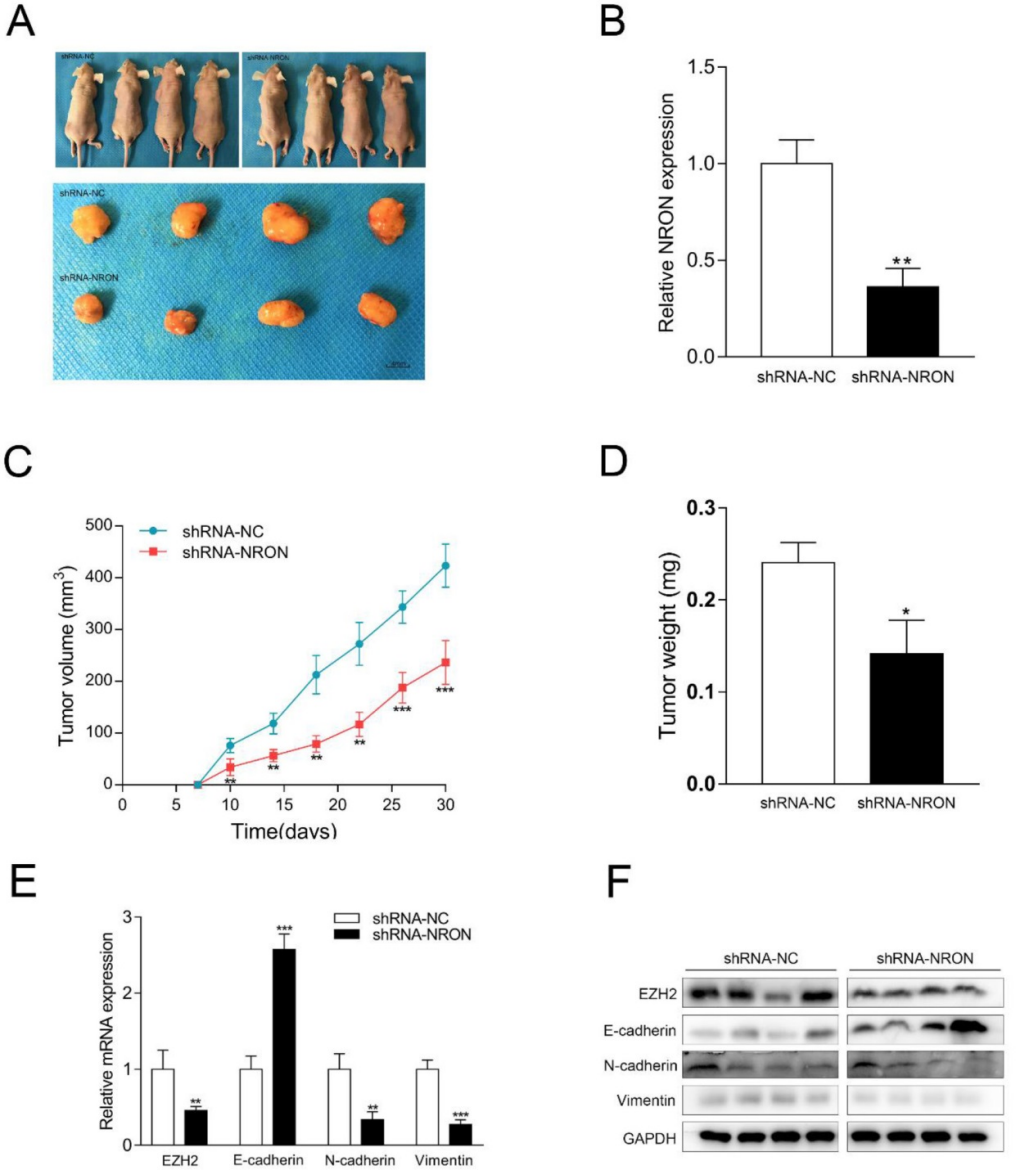
NRON promotes tumorigenicity of BC cells. (A) Tumors collected from mice were exhibited. (B, C) The weight and volume of tumors from mice were measured and analyzed. (D) The level of NRON expression in tumors from mice was tested by RT-qPCR. (E, F) Knockdown of NRON decreased EZH2 expression and inhibited EMT of BC cells* in vivo*. (*P < 0.05, **P < 0.01, ***P < 0.001).

We used a subcutaneous xenograft mouse model to assess the effect of NRON on tumor growth *in vivo*. The SW780 cells with sh-NRON or sh-NC were subcutaneously injected into the flanks of balb/c nu-nu mice. Four weeks after injection, compared with control groups, sh-NRON group displayed significant reduction in both tumor volume and weight (Fig. 5A-D). These data suggest that NRON promotes tumor growth of BC *in vivo*.

Thereafter, we also analyzed the expression patterns of NRON, EZH2 and EMT markers in these tumor samples from nude mice. The mRNA level of NRON in the sh-NRON group was reduced by 63.7% compared to the control group (Fig. 5B). Similar to our *in vitro* results, knockdown of NRON led to upregulation of E-cadherin, and downregulation of Vimentin and N-cadherin (Fig. 5D and E). Moreover, knockdown of NRON significantly decreased EZH2 expression (decreased by 54.2%) (Fig. 5D). These results suggest that NRON promotes EMT progression, and NRON-induced EZH2 expression contributes to this process.

## Discussion

LncRNA play various roles in the regulation of transcriptional activation, X chromosome inactivation, heterochromatin formation, and maintenance of telomeres 47-49. LncRNA are considered as oncogenes or tumor suppressors in cancers. For instance, lncRNA metastasis-associated lung adenocarcinoma transcript 1 (*MALAT1*) has been reported to be upregulated in various cancers and act as an oncogene in breast cancer 50, 51. Additionally, *MALAT1* regulates the expression of N-cadherin and E-cadherin in BC 52-54. On the contrary, NF-κB Interacting lncRNA (*NKILA*) is an example of tumor suppressor gene 55. *NKILA* acts as a NF-κB regulator to inhibit breast cancer metastasis 56-58.

Previous studies have highlighted the potential of lncRNA as biomarkers and therapeutic targets in BC. LncRNA NRON (location: 9q33.3) acts as an T-cell nuclear factor (*NFAT*) repressor 45. NRON inhibited *NFAT* activity and inhibited the proliferation and invasion of vascular endothelial cells through reducing *NFAT* nucleus transfer 38. Moreover, it is reported that HIV-1 promoted infectivity through utilizing NRON 39. NRON has been shown to be downregulated in HCC and NRON downregulation is associated with poor clinical prognosis for HCC. NRON overexpression inhibits the EMT process, thereby inhibiting HCC growth and metastasis 44. However, the role and underlying mechanisms of NRON in BC is not clearly understood.

To the best of our knowledge, this study reports for the first time that NRON is upregulated in BC tissues and cell lines and demonstrates the function of NRON in BC. In the present study, we revealed that the upregulation of NRON in bladder cancer patients was significantly associated with the depth of bladder tumor invasion and poor prognosis. Knockdown of NRON inhibited BC cells proliferation, migration, invasion and tumorigenicity. Furthermore, NRON promoted epithelial-mesenchymal transition (EMT) progression, and NRON-induced EZH2 expression contributed to this process. Our results provide important clues regarding NRON as an oncogene and promising tumor biomarker for BC.

EMT is an important biological process in cancer development, in which epithelial cells lose their cell adhesion properties and are transformed into a mesenchymal phenotype 59. The process of EMT promotes multiple tumor cells to gain the capacity to infiltrate adjacent normal tissues and ultimately to metastatic spread 60. EZH2 is the catalytic subunit of polycomb repressive complex 2 (PRC2), which can epigenetically regulate gene expression 61. A large number of evidences revealed that EZH2 serves as a critical role in carcinogenesis 62. Previous reports showed that EZH2 promotes EMT 63, and EZH2 can directly induce EMT or indirectly initiate EMT 64, 65. In addition, several miRNAs have been reported to regulate EMT via EZH2 66. In this study, NRON-induced EZH2 expression promoted the progression of EMT, thereby promoting invasion and metastasis of BC cells.

NRON is down-regulated in HCC and overexpressions of NRON suppress HCC growth and metastasis 44. NRON was also down-regulated in triple-negative breast cancer (TNBC), and NRON down-regulates lncRNA snaR to inhibit TNBC cell proliferation 46. In this study, we showed that NRON is upregulated in BC tissues and cell lines, and the upregulation of NRON in bladder cancer patients was significantly associated with the depth of bladder tumor invasion and poor prognosis. We now know that the same gene can act as tumor suppressor in one cell type and as potential oncogene in other cell types, such as H19 or P53 67-70. Because lncRNA expression patterns are highly specific for cell type and cell differentiation status, and the function of lncRNA depends on the combination of interaction partners in the respective cancer cells that execute the function of lncRNA. NRON showed different expression patterns between BC and HCC, suggesting that NRON expression is highly specific for cell types. NRON is likely to have different functions in different cancer cells. Interactions with other protein or even other nucleic acids (e.g miRNAs, mRNAs, ncRNAs, or DNA) could be possible and would increase the spectrum of NRON functions. Although a series of experiments have been used to explore the functions of NRON in BC in this study, further researches of NRON were still needed. Further research is needed to elucidate the molecular mechanisms by which NRON regulates proliferation, migration, invasion, and EMT of BC cells.

## Figures and Tables

**Table 1:** Correlation between NRON expression and clinicopathological characteristics of bladder cancer patients.

Characteristics	Total	Expression of NRON		*P* value
		High (n=36)	Low (n=6)	
**Gender**				
Male	36	32 (88.9%)	4 (11.1%)	0.150
Female	6	4 (66.7%)	2 (33.3%)	
**Tumor size (cm)**				
<4 cm	19	17 (89.5%)	2(10.5%)	0.527
≥4cm	23	19 (82.6%)	4 (17.4%)	
**Age**				
≤60	22	21 (95.4%)	1 (4.5%)	0.058
>60	20	15 (75%)	5 (25%)	
**Tumor invasion depth (T)**				
Tis, Ta, T1	35	32 (91.4%)	3 (8.6%)	0.018
T2, T3 or above	7	4 (57.1%)	3 (42.9%)	
**TNM stage**				
0/I	28	23 (82.1%)	5 (17.9%)	0.350
II/III/IV	14	13 (92.9%)	1 (7.1%)	

TNM according to the seventh edition of staging TNM of Union for International Cancer Control (UICC) in 2009.
